# Caseload midwifery as organisational change: the interplay between professional and organisational projects in Denmark

**DOI:** 10.1186/s12884-015-0546-8

**Published:** 2015-05-27

**Authors:** Viola Burau, Charlotte Overgaard

**Affiliations:** CFK - Public Health and Quality Improvement, Olof Palmes Allé 15, 8200 Aarhus N, Denmark; Department of Political Science, University of Aarhus, Bartholins Allé 7, 8000 Aarhus C, Denmark; Department of Health Science and Technology, Aalborg University, Fredrik Bajers Vej 7D, 9220 Aalborg, Denmark

**Keywords:** Caseload midwifery, Organisational change, Denmark, Professional project, Management

## Abstract

**Background:**

The large obstetric units typical of industrialised countries have come under criticism for fragmented and depersonalised care and heavy bureaucracy. Interest in midwife-led continuity models of care is growing, but knowledge about the accompanying processes of organisational change is scarce. This study focuses on midwives’ role in introducing and developing caseload midwifery. Sociological studies of midwifery and organisational studies of professional groups were used to capture the strong interests of midwives in caseload midwifery and their key role together with management in negotiating organisational change.

**Methods:**

We studied three hospitals in Denmark as arenas for negotiating the introduction and development of caseload midwifery and the processes, interests and resources involved. A qualitative multi-case design was used and the selection of hospitals aimed at maximising variance. Ten individual and 14 group interviews were conducted in spring 2013. Staff were represented by caseload midwives, ward midwives, obstetricians and health visitors, management by chief midwives and their deputies. Participants were recruited to maximise the diversity of experience. The data analysis adopted a thematic approach, using within- and across-case analysis.

**Results:**

The analysis revealed a highly interdependent interplay between organisational and professional projects in the change processes involved in the introduction and development of caseload midwifery. This was reflected in three ways: first, in the key role of negotiations in all phases; second, in midwives’ and management’s engagement in both types of projects (as evident from their interests and resources); and third in a high capacity for resolving tensions between the two projects. The ward midwives’ role as a third party in organisational change further complicated the process.

**Conclusions:**

For managers tasked with the introduction and development of caseload midwifery, our study underscores the importance of understanding the complexity of the underlying change processes and of activating midwives’ and managers’ interests and resources in addressing the challenges. Further studies of female-dominated professions such as midwifery should offer good opportunities for detailed analysis of the deep-seated interdependence of professional and organisational projects and for identifying the key dimensions of this interdependence.

## Background

The large obstetric units in which most births take place in industrialised countries are often criticised for their fragmented and depersonalised care in highly bureaucratic settings [1–3]. With mounting evidence of improved clinical outcomes and care satisfaction in pregnant women [4–8], interest in midwife-led continuity models of care is growing.

Yet, as Forster et al. have remarked, studies to guide the introduction of midwife-led models of care are few and far between [9]. Two Australian studies have explored the specific processes and contexts underpinning the organisational processes involved. In Walker et al. it is reported that organisational structures may be both facilitators and obstructions to the implementation [10]. Management support, professional development and teambuilding exercises emerged as key factors. Hartz et al.’s study points to the importance of midwives’ relationships with other health professions [11].

We follow Forster and colleagues [9] in insisting that a theoretical framework is key to understanding organisational change processes in complex interventions such as caseload midwifery. As midwives’ dissatisfaction has been identified as the main reason for reverting to more traditional models of care [9], we focus on their involvement in organisational change. Sociological studies of midwifery and recent contributions to organisational studies of other professions are used to frame the analysis. This helps to capture midwives’ strong interests in caseload midwifery and their key role in negotiating organisational change with management.

Sociological studies of the midwifery profession suggest that caseload midwifery is likely to appeal to midwives’ professionalism [12–15]. This model of organising midwifery services contrasts sharply with standard care in specialised hospital settings; the continuity of carer throughout pregnancy, childbirth and the postpartum period supports a highly individualised service [16] in which individual midwives, or a small group of midwives, care for their own caseload of women [8, 17, 18], thus allowing for a high level of autonomy in professional practice [19]. Caseload midwifery may be seen as a strong professional project as it presents a key opportunity to strengthen midwifery as a profession [13, 20].

Midwives’ professional interest in the caseload model may be supported by policy-makers’ and administrators’ concurrent aim to reform and develop health services [12, 14, 20–22]. This can be thought of an ‘organisational project’. The development of female-dominated professions such as midwifery has gone hand-in-hand with building the healthcare state [12, 14]. At present, caseload midwifery coincides with a concern for more patient-centred and efficient health services [23, 24] and in England, for example, the government was key in promoting this new model of care [13].

Recent contributions to organisational studies of professions [25–28] help to specify the interplay between professional and organisational projects in caseload midwifery; they focus on the connections between midwives’ professional work and hospital management’s organisational activities. The literature maintains that the interests of professional groups and management together form a complex pattern to control hospitals and midwifery practices, including professional control mechanisms such as peer review and managerial control mechanisms such as quality audits. Muzio and colleagues and Noordegraaf [29–31] introduce the idea that rather than existing side by side, professional and organisational projects are interdependent, thus indicating that midwives are important actors in caseload midwifery as an organisational project, as much as hospital management contribute to the development of caseload midwifery as a professional project.

Against this background, we analysed how midwives and management, with the resources at their disposal, engaged in micro-processes of organisational change in the pursuit of their respective interests. Assuming an interdependence between midwives’ and management’ interests and resources, we focused on the hospital as the shared organisational platform for their negotiations, and also considered the broader contexts of change [26].

Denmark was chosen because of its long tradition for midwifery and government support of midwives’ autonomy in care for women with normal pregnancies/births, dating back to the 18th century [32]. With relatively low rates of medical intervention in childbirth, the Danish debate on maternity care has focused less on the medicalisation of birth than on growing bureaucracy and workloads at increasingly large and busy obstetric units and on how this potentially undermines care continuity and midwives’ work satisfaction [33, 34]. Across the country, caseload midwifery is the norm in maternity wards, thus offering an opportunity to study caseload midwifery as a strong professional and organisational project characterised by an intense interplay among actors and across their respective organisational/professional interests and resources.

This study specifically aimed to explore:

- The interplay between midwives and management in their negotiations on the introduction and development of caseload midwifery
- The professional and organisational interests pursued by midwives and management in the process
- The professional and organisational resources activated by midwives and management in the process.

## Methods

We used a qualitative multiple-case design in which a case was defined as a hospital with a chief midwife and her deputy and an autonomous organisation of midwifery services in which caseload midwifery had been introduced less than a year before the spring of 2013. This choice was motivated by the wish to capture both the introduction and the subsequent development of caseload midwifery through practice.

### Conceptual framework of the analysis

The framework of the analysis had three components: processes, interests and resources.

Processes concern the negotiations on the introduction of caseload midwifery and its development through practice. We use May and Finch’s [35] general definition of organisational change as the social organisation of implementing new modes of action. This takes place in several iterative processes that involve both formal and informal negotiations, for example establishing local collective agreements and midwives’ and management’s day-to-day negotiations.

Midwives and managers pursue interests and command resources, and we expect each group to pursue its own both professional and organisational interests by applying its resources. A relevant indicator for midwives’ interests is to what extent they see caseload midwifery to be in line with their professional interests in the development of care and in good working conditions (similarly [36, 37]). A relevant indicator of management’s interests is the organisational aims connected with caseload midwifery, and to what extent they co-exist with interests in furthering midwives’ professional development. Midwives and managers can also draw on different types of resources when negotiating caseload midwifery: Midwives can rely on their specialised and practical knowledge as well as on their rights as employees in a unionised workplace; managers command both hierarchical power and “softer” forms of power arising from their shared professional background with staff midwives.

### Selection of cases

The selection of hospitals aimed at maximising variance and thus the robustness of our findings [38]. Three units were identified on the basis of the type of hospital and their caseload model (see Table 1).

**Table 1:** Overview of cases

Type of hospital	Caseload model Funding	Scale of model	Caseload midwifery targets	Caseload details*
*Highly specialised university hospital*	Funded by reduced staffing of ward midwives from:	4 caseload groups	Nulliparas + women who plan early discharge + planned homebirths in hospital catchment area (1 %)	• 120 births per annum per group
	8 a.m.		(1 group with 2 midwives, 3 groups with 3 midwives)	• Mixed risk status
Obstetric unit with 4900 births	8 p.m.			• Max 50 % nullipara
	7 p.m-7 a.m.			
7 p.m.				
	Neonatal intensive care unit			
	7 p.m.			
*Specialised mid-level hospital*	Funded by reduced staffing of ward midwives from:	8 caseload groups	Nulliparas	• 120 births per annum per group
Obstetric unit with 2400 births	6 a.m.		(6 groups: 1 with 2 midwives, 5 with 3 midwives)	• Mixed risk status
	6 p.m.			• 100 % nullipara
	5 a.m-5 p+a.m.		Vulnerable and/or socially dis-advantaged mothers **	• 120 births per annum per group
Neonatal intensive care unit	5 p.m.		(1 group with 3 midwives)	• Mixed risk status
	4 a.m.		Twin pregnancy or women with fear of childbirth	• Mixed nulli- and multiparas
			(1 group with 2 midwives)	
*Community hospital*	Earmarked funding for pilot project	2 caseload groups	All women from local area	• 140 births per annum per group
Obstetric unit with 1900 births			(2 groups, each with 3 midwives)	• Mixed risk status
No neonatal intensive care unit				• Mixed nulli- and multiparas

*Groups consisted of two full-time midwives (37 h/weekly average) or three midwives working either part-time (e.g. 30 h/week) or full-time, divided between caseload (e.g. 25 h/weekly average) and ordinary ward shifts (e.g. 12 h/weekly average)

**Pregnant women, e.g. who are young (<20 years) and/or affected by mental health or social problems

The three hospitals shared overall guidelines for midwifery practice; the number of midwives and women in the caseloads were similar, as was the time of introduction of caseload midwifery (2012/13). However, the hospitals varied in theoretically important respects and illustrated very different conditions for introducing and developing caseload midwifery. Three different types of hospital were represented (university, mid-level and community hospital), their number of annual births varied (4900, 2400 and 1900, respectively) and there were differences in terms of the scale and funding of the caseload model. At the community hospital, the introduction of caseload midwifery in two teams was externally funded as a pilot project. In the two other cases, funding was found by reducing the staffing level among ward midwives with the equivalent of one shift. The scale of change was largest at the mid-level hospital where eight caseload groups were established.

The target groups for caseload midwifery also varied, thus highlighting differences in the local conditions for introducing and developing caseload midwifery. The community hospital focused on all birthing women in their area, resulting in highly diverse caseloads. The larger hospitals defined their primary target groups as first-time mothers and potentially very labour-intensive births given by vulnerable/socially disadvantaged women, or women with special needs.

### Data collection

Data were generated from semi-structured interviews with key respondents; this occurred individually with the chief midwife and her deputy, and on a group basis with the caseload/ward midwives, obstetricians and community health visitors. The recruitment of respondents aimed to maximise variance through a purposive approach to sampling. In the mid-level hospital the group working with vulnerable mothers was included; in relation to all other informants, access to the widest possible range of experiences was prioritised. The researchers were assisted by the chief midwife or her deputy in identifying caseload groups and individual informants for invitation to participate.

We intended to interview the three sets of managers individually while we felt small groups were most suitable otherwise to stimulate reflection and discussion among participants that were confident with each other and thus could provide richer data. However, interview scheduling with caseload midwives proved particularly difficult and some of them had to be interviewed individually. We conducted 10 individual and 14 group interviews.

The interviews lasted 30–40 min and were conducted in spring 2013. Based on the conceptual framework an interview guide was developed to cover the following five themes: (1) the local caseload model, (2) introduction processes, (3) development processes, (4) professional interest in caseload midwifery and (5) collaboration with other professional groups/caseload midwives.

### Ethics

Danish legislation requires no ethical approval for this type of study (see Act on Research Ethics Review of Health Research Projects, Law no. 593, 14 June 2011; http://www.cvk.sum.dk/English/actonabiomedicalresearch.aspx). The specific guidelines on qualitative studies state that ‘questionnaire-based examinations shall be treated like the so-called register research projects, i.e. that they have to be notified ONLY if the project will include examination of human biological material or examination of individuals (…) Interview examinations are comparable to questionnaire-based examinations’ (Section 2.8, Guidelines about Notification etc. of a Biomedical Research Project to the Committee System on Biomedical Research Ethics, No 9154, 5 May 2011; http://www.cvk.sum.dk/English/guidelinesaboutnotification.aspx)

All participants were thoroughly informed about the study before their written consent was obtained. All interviews were recorded and transcribed verbatim. Participants were given the possibility of adding to and deleting from the original transcript of the interview. Direct or indirect references to the individual hospitals and the individual participants were subsequently removed. All participants approved the final written and anonymised transcript. As the study involved identifiable high-profile participants, Table 2 and the quotes contain only basic information about the participants.

**Table 2:** Overview of interviews and participants

Case site	University hospital	Mid-level hospital	Community hospital
Individual interviews	*Chief midwife*	*Chief midwife*	*Chief midwife*
	*Deputy chief midwife*	*Deputy chief midwife*	*Deputy chief midwife*
	*Obstetrician* ^*,**^	Caseload midwifery group C***	Caseload group G***
	• One obstetrician > 10 years’ experience	*[2 midwives working with first-time mothers]*	• One midwife > 10 years’ experience
		• One midwife < 5 years’ experience, One midwife > 10 years’ experience	
Group interviews	*Caseload group A*	*Caseload group D*	*Caseload group F*
	*[3 midwives working with primiparas + women planning early discharge + all planned homebirths in hospital catchment area]*	*[3 midwives working with vulnerable/socially disadvantaged mothers]*	*[3 midwives working with all women in a specific geographical area]*
	• Two midwives > 10 years’ experience	• One < 5 years’ experience	• One < 5 years’ experience
	* Total: 2 participants*	Two > 5 years’ experience	Two > 10 years’ experience
		* Total: 3 participants*	* Total: 3 participants*
	*Caseload group B*	*Caseload group E*	*Caseload group G*
	*[2 midwives working with primiparas + women who plan early discharge + all planned homebirths in hospital catchment area]*	*[3 midwives working with first-time mothers]*	*[3 midwives working with all women in a specific geographical area]*
	• One < 5 years’ experience	• Two < 5 years’ experience	• One < 5 years’ experience
	One > 10 years’ experience	* Total: 2 participants*	One > 10 years’ experience
	* Total: 2 participants*		* Total 2 participants*
	*Ward midwives*	*Ward midwives*	*Ward midwives*
	• One < 5 years’ experience,	• Two < 5 years’ experience	• Two > 5 years’ experience
	Two > 5 years’ experience	One > 5 years’ of experience	One > 10 years’ experience
	* Total: 3 participants*	One > 10 years’ experience	* Total: 3 participants*
		* Total: 4 participants*	
	Health visitors*	Obstetricians*	Obstetricians*
	• One < 5 years’ experience,	• One < 5 years’ experience	• One < 5 years’ experience
	One > 5 years’ experience	One > 5 years’ experience	One > 5 years’ experience
	One > 10 years’ experience	One > 10 years’ experience	One > 10 years’ experience
	* Total: 3 participants*	* Total: 3 participants*	* Total: 3 participants*
		Health visitors*	Health visitors*
		• One > 5 years’ experience	• Two > 10 years’ experience
		Two > 10 years’ experience	One student
		* Total: 3 participants*	* Total: 3 participants*

*Very limited reporting of data from interviews with obstetricians and health visitors in this analysis. The interviews showed that other professional groups played an extremely limited role in the negotiation process, possibly because the introduction of caseload midwifery involved no significant changes in the distribution of tasks among midwives and related professionals

**Obstetricians single-handedly decided that an individual interview with key representative of the group was most appropriate due to limited knowledge of the introduction of caseload midwifery among obstetricians in general and work pressures at time of data collection

***Practical conditions dictated that interviews were individual

### Data analysis

The analysis began by constructing and applying a set of codes derived from the operationalisation of the conceptual framework. Using NVivo 10 software we analysed the interview material based on a thematic approach that combined deductive and inductive elements aiming at identifying common threads [39]. The resulting codes were then collated to create preliminary themes, which were subsequently reviewed and refined. We first conducted a within-case analysis, followed by a cross-case analysis. We did this both individually and jointly, and the iterative nature of the work resulted in a truly joint analysis.

## Results

The following analysis is based on individual and group interviews with a total of 49 study participants. Table 2 provides selected information on the individuals involved.

The study participants included different occupational groups (midwives, obstetricians, health visitors) and different groups within midwifery (midwife manager, caseload midwife and ward midwife). The individual participants had different levels of professional experience.

### Negotiating the introduction of caseload midwifery

#### Processes

Formal processes dominated the introductory phase, including initial decision-making on the introduction of caseload midwifery, the recruitment of midwives, assembling the individual groups and negotiating the local collective agreement. In theory this gave management the upper hand, but in practice the midwives also wielded influence; the decision to join caseload midwifery had to be voluntary as it presented a change in working conditions. Management, especially at the community and the university hospital, gave priority to offering a real choice. As the deputy chief midwife at the university hospital explained, the decision to join caseload midwifery would have far-reaching implications for their private lives, as midwives would typically be on call at all hours for seven days.

In contrast, organising caseload groups took place in informal negotiations among the involved midwives. Two midwives from the community hospital expressed a general feeling of freedom to organise the group in a way that suited them best, provided that the collective agreement was respected. The practical issues at hand included preparing duty rosters, establishing a system for booking appointments and writing information leaflets. Detailed guidance was neither found necessary nor desirable. The complexity of the organisational issues stemmed in part from the close collaboration necessary for the small groups to function well.

#### Interests

The management was driven by both organisational and professional interests in the negotiations on the introduction of caseload midwifery. Organisational interests stemming from concurrent changes at the individual hospital were apparent for example at the community hospital. There was a strategic interest in attracting birthing women from the local area as the centralisation of maternity care units had increased competition between the region’s units. However, the management also formulated professional interests in the development of midwifery; one of the chief midwives thus said:

*… a holistic approach and continuity are the core values of midwifery – and they are key reasons for introducing caseload midwifery ….* (chief midwife, university hospital)

Similarly, the midwives pursued both professional and organisational interests in the negotiations. The latter were reflected in their strong wish to ensure clarity concerning recruitment and working conditions. For example, a mid-level hospital midwife emphasised confusion about management’s attitude to midwives’ initial interest in caseload midwifery and the resistance this had created.

*Not much time passed between the idea of caseload midwifery was introduced …, something that we as midwives felt part of, and the decision was taken. … there were many who fought back a little.* (caseload midwife, mid-level hospital)

As the following quote shows, the wish for clarity also concerned a broader, underlying interest in a well-defined framework for future work:

*[Introductory meetings] were arranged on short notice and … many could not attend. … However, [the information] was always provisional, because [the caseload model] had to be adapted in the ways [the individual group] considered appropriate. So sometimes we couldn’t get an answer.* (ward midwife, community hospital)

Finally, clarity about working conditions stemmed from the midwives’ interest in a good work-life balance enabling them to combine work with family responsibilities. As a midwife from the mid-level hospital explained, many were doubtful about how they would be able to square the circle.

The midwives’ interests in clear work regulations were coupled with an equally strong interest in caseload midwifery as an opportunity for professional development. They were attracted by the opportunity for greater continuity and improving quality. The following quote is typical:

*We all felt that [the greater continuity] would benefit the women – that when they came in to give birth, they would have a midwife that they knew.* (caseload midwife, community hospital)

For the midwives, caseload midwifery also promised greater professional satisfaction, with a more holistic approach corresponding with the core tenets of their professionalism. A midwife said:

*But you know, I have always been strongly committed to normal birthing … and the meeting with the family. …. caseload midwifery was a great opportunity to work in a more“original” way.* (caseload midwife, university hospital)

Yet, professional and organisational interests can be contradictory. According to the chief midwife in the university hospital, caseload midwifery combined the “employee model” and the “self-employment model” and presented an important challenge for the introduction and development of caseload midwifery.

#### Resources

Managers and midwives drew on both organisational and professional resources. In all three hospitals management invoked hierarchical resources to secure the decision to introduce caseload midwifery, whereas only the mid-level hospital used hierarchical resources to secure recruitment. As its chief midwife explained, too few midwives were interested in joining caseload midwifery, forcing management to fill the gap with midwives whose temporary contract was up for renewal.

Nevertheless, management also used professional resources by appealing to shared professional interests. For example, the university hospital midwives’ felt that their uncertainty about the implications of caseload midwifery was not accommodated. As a result, the local collective agreement was signed only a few days before the deadline. The chief midwife described her efforts to assuage the worries:

*We spend a lot of time calming down and building trust, saying that “if this does not work, we’ll simply stop. You won’t be locked up in [caseload midwifery] in eternity”.* (chief midwife, university hospital)

The midwives drew on both organisational and professional resources in negotiating the introduction of caseload midwifery. The fact that the introduction of caseload midwifery rested on their adoption of a new collective agreement gave them organisational resources, as was forcefully illustrated by the stressful last days before the groups started up at the university hospital. In contrast, professional resources came into play especially when the individual caseload midwifery groups were organised. The midwives’ position was strengthened by their proximity to practice, which allowed them to rely on their knowledge of previous practice as well as on very early experiences with caseload midwifery. For example when one group in the university hospital decided to move from half-week shifts to full-week shifts after a few months.

### Negotiating the development of caseload midwifery through practice

#### Processes

Allowing women the opportunity to be followed by the same midwife all along represents a cornerstone in caseload midwifery. Negotiating the concept of continuity and its practical implications was therefore a key issue. Although dominated by informal processes, the negotiations were complemented by formal reviews of experiences and caseload sizes.

#### Informal processes

The development of the caseload models required group members to agree on procedures for the documentation of care, follow-up and booking arrangements as well as on the collaboration with ward midwives. Standards for continuity and workload were continuously under discussion as the different caseload models each appeared to have specific strengths and weaknesses. Caseload groups of two and those working with women with special needs succeeded in achieving good continuity, but some midwives felt that the quality of work and their personal work satisfaction were challenged by the frequent and long duty turns. A model involving three midwives appeared to offer more personal flexibility but poorer continuity of care.

Regulations on day-to-day collaboration among caseload and ward midwives created a need for negotiations at the three hospitals. Issues included the specifications in the collective agreement on rest hours after long duty turns, the number of monthly on-call duties and peak-time assistance in the labour ward. Both caseload and ward midwives had difficulties with assessing when asking for and offering help was justified:

*I think the ward midwives find that our phones are turned off a lot [while caseload midwives rest] and that they are frustrated by that. It is frustrating to ask for help when you have been working for many hours and you’re really tired and … no one has time to help you.* (caseload midwife, mid-level hospital)

Danish labour wards are staffed primarily by registered midwives, who enjoy high levels of autonomy and share tasks and working conditions, but the division into ward midwives and caseload midwives disrupted this. In all three hospitals, the caseload midwives were said to be “running their own business” and thus no longer full members of the ward’s working community. Frictions were felt particularly strongly in the busy labour wards at the two larger hospitals. Tensions were possibly further fuelled by the fact that introduction of caseload midwifery had been funded by a reduction in the permanent staffing of ward midwives.

#### Formal processes

Negotiations also followed more formalised paths. Evaluations were conducted at all three hospitals, but otherwise the arrangements differed. As the largest unit, the university hospital had the most extensive and systematic set-up with monthly meetings between caseload groups and management. Caseload midwives supported this approach:

*Management have been very visible and made sure that we meet regularly. … We feel they support the model but also acknowledge it’s important that we’re okay.* (caseload midwife, university hospital)

At the other hospitals, regular meetings were still being considered, and in some instances midwives felt that management had become less accessible than before. For example, the caseload groups at the mid-level hospital experienced serious problems with their caseload of only first-time and/or vulnerable mothers but found it difficult to make their views heard by management. Their frustration led them to organise a formal meeting with the chief midwife to discuss their experiences.

#### Interests

As already mentioned, a division of midwives’ interests arose, but the respective professional and organisational interests were closely intertwined, when the development of caseload midwifery was negotiated.

The caseload midwives felt the new model fulfilled their professional ambitions and expectations, and saw it as a way to improve both medical and psychosocial standards. Their strong sense of duty towards the women was expressed by a midwife caring for vulnerable mothers:

*They [the pregnant women] need security. During pregnancy we promised them [to be there during birth] and we must fulfil that promise.* (caseload midwife, mid-level hospital)

Professional interests coexisted with an organisational interest in a caseload model that allowed for a good work-life balance. This became particularly apparent in situations, where the two interests pulled into opposite directions, as it did in particular at the mid-level hospital, where some caseload midwives experienced a strong work overload:

*As someone said, if you prioritise professional issues, choose a two-in-group model – if you prioritise family, choose a three-in-group model. I was hoping one could unite interests in a three-in-group model.* (caseload midwife, mid-level hospital)

Contextual factors may have been important here. At the mid-level hospital, negotiations on the caseload models were conducted among staff who were hardly ready for further changes as they were still grappling with the effects of a recent, comprehensive organisational upheaval and budget reductions.

The interests of ward midwives emerged from a two-fold view that caseload midwifery was very much organised independently of the ward and that caseload midwifery offered greater continuity. Recent developments may have further accentuated this view as the introduction of caseload midwifery at the two larger hospital had occurred at the expense of staffing levels among ward midwifes, which may have created tensions between the two groups.

The caseload midwives were concerned about the perception of a dichotomy between those who were supposed to deliver continuity and individualised high quality care and those delivering standard, potentially fragmented and routine “factory line” care. A caseload midwife pointed out:

*Some [ward midwives] feel they are the bad choice. Because … [caseload midwifery] is oh, so good and so positive. But they [ward midwives] are also doing a good job. When they deliver one of ours, because our phone is turned off, they worry that they are not performing well enough.* (caseload midwife, mid-level hospital)

This situation seemed to have affected some of the ward midwives to strive for higher standards despite the limited continuity offered by their traditional model.

The organisational interests of ward midwives stemmed from their uncertainty about the overall effects of introducing caseload midwifery. Some expressed their sense that workloads had increased and that caseload midwives had highly privileged working conditions. This fuelled their organisational interests in ensuring a fair distribution of work among ward and caseload midwives. Some of the ward midwives expected caseload midwives to take complete responsibility for women in their caseload:

*If they [the pregnant women in caseload groups] are going to get to know that midwife, then they must see her, also for the trivial problems.* (ward midwife, mid-level hospital)

Concerns over increasing divisions between the two groups were also at the centre of management’s organisational interests. In all three hospitals they spoke repeatedly of the necessity of ensuring unity. A chief midwife said:

*When you [the caseload midwives] are on the ward and she [the labouring women in her caseload] is in early labour – go and offer your help! .... It is important that they [the caseload midwives] do something to show the other midwives that we are all working together.* (chief midwife, university hospital)

Management’s interest in maintaining a strategic focus on the development of the organisation as a whole coincided with economic interests. In times of austerity, management has a strong interest in cost efficiency and keeping expenses under control. The following quote is typical:

*You can be sure that if I do not believe we improve quality substantially or use resources in a more effective way, then I won’t agree to it. If this [caseload midwifery] costs money that I don’t have, then that’s going to be a major issue!* (chief midwife, university hospital)

Nevertheless, for management in all three hospitals, professional interests were important drivers in the negotiation process. These interests emerged as the principal rationale for caseload midwifery, as underlined below by the chief midwife’s focus on maintaining work satisfaction among caseload midwives.

*… I actually experience midwives saying that they do not disagree with the professional goal in this [caseload midwifery]. They agree that our focus on first-time mothers is unique. It makes sense, professionally .... We just have to adjust [the caseload size] because otherwise they can’t sustain their work satisfaction.* (chief midwife, mid-level hospital)

#### Resources

Also in negotiations on the practical development of caseload midwifery, midwives and management were found to draw on both professional and organisational resources.

Midwives drew on their professional resources and practical experience, especially in organising their groups, the planning of care and resolving practical issues. These resources included their professional knowledge as well as their experience from related work.

In case of uncertainty, the caseload midwives extended their evidence base by including performance data from the maternity unit to further strengthen their resources. For example, in the mid-size hospital, a management report on caseloads and on-call duty turns played an important role in the negotiations by documenting in “management-speak” the issues identified by the caseload midwives. This helped caseload midwives make themselves heard:

*It isn’t actually until now that numbers have been added, … that [the large workload] has been taken seriously. Because I have talked to the head earlier … and she said, “Well, it [the workload] always varies a bit, it will always be like that”.* (caseload midwife, mid-level hospital)

The midwives also activated organisational resources derived from the collective agreement. For example, the university hospital midwives asked the local shop steward to help them resolve questions about the interpretation of rest and on-call duty regulations.

The management at all three hospitals also drew on a combination of professional and organisational resources. Professional resources were involved in the frequent appeals to unity among midwives in negotiations on the caseload model. The unit was always constructed as one organisation, most clearly expressed by the mid-level hospital chief midwife:

*We are one unit. And that is my managerial focus at staff meetings and the like. I constantly make sure that I contribute to the creation of a sense of community [in the unit]. Because I think this is one of the risks related to caseload midwifery.* (chief midwife, mid-level hospital)

Management had two types of organisational resources at its disposal. Hierarchical authority was used mainly in the negotiations on caseload sizes and the responsibilities involved. For example, after a meeting organised by the caseload midwives at the mid-level hospital, the chief midwife agreed to a slight reduction of caseload sizes. Management also drew extensively on softer technical resources such as staff statistics and managerial reports. As the report was updated every three months, it helped management to survey developments and identify problems requiring action.

## Discussion

### Mapping organisational change processes in caseload midwifery

This paper set out to expand the understanding of organisational changes involved in caseload midwifery. Our theoretical argument that change processes are characterised by a close interplay of professional and organisational projects was corroborated by the analysis, which identified three indicators of this interdependence.

Firstly, both the introduction and the development through practice were dominated by formal and informal negotiations, with variations in the balance between them. Formal negotiations dominated the introductory phase, i.e., the initial decision, the recruitment of midwives, group establishment and completing the collective agreement, although more informal negotiations on group organisation were also observed. In contrast, informal negotiations prevailed in the development through practice, such as establishing procedures for follow-up and booking, in-group coordination of tasks and offering help to or asking for help from the other midwives. This was complemented by more formal negotiations centred on the review of the caseload.

Secondly, midwives and managers engaged in both types of projects, i.e., they each pursued professional as well as organisational interests in caseload midwifery, and in doing so, they drew on professional as well as organisational resources. Occasionally, this happened in tandem while at other times, this occurred in a sequence. The process leading to the local collective agreement at the university hospital provided an example of the complex dynamics. The midwives were drawn between a concern that working conditions remained unclear and their enthusiasm about the caseload model, which they welcomed as an opportunity to work in a professionally more meaningful way. In the ensuing negotiations with management, the midwives combined professional and organisational interests by withholding their support for the agreement when they invoked organisational resources as employees in a unionised workplace. The situation caused management to put aside its organisational interests and resources and instead appeal to the common interest in good working conditions for the future caseload midwives and emphasise its interests in caseload midwifery as an opportunity for professional development.

Thirdly, as caseload midwifery was deeply embedded as both a professional and an organisational project, the two parties strained to resolve any tensions between these projects. For example, in the negotiations on the caseload size in the mid-level hospital, the midwives saw the existing caseload as a trade-off between their professional and organisational interests and felt they could only provide good continuity if they compromised on their own work-life balance and accepted long duty calls. While management was initially unreceptive to their concerns, the situation was changed when a combination of professional and organisational resources were invoked. The midwives compiled their experiences with caseloads across groups and supported them with performance data. In response, the chief midwife focused on her interest in safeguarding the professional stakes related to caseload midwifery and used “soft” organisational resources in the form of performance data in the negotiations. The parties reached a compromise on a minor reduction of the size of caseloads.

It is interesting to note how the interplay between professional and organisational projects in the introduction and development of caseload midwifery was complicated by ward midwives’ role as a third party. They defended distinct professional and organisational interests by insisting, despite the lower degree of continuity on their wards, on high standards and a fair distribution of work vis-a-vis the caseload midwives. In turn, maintaining the unity among midwives as a whole became a main organisational interest for management.

### Methodological considerations

In generalising the results, it should be taken into account that our study involved only three hospitals, all in the same region of Denmark. The local context was highly influential in the organisational change processes in caseload midwifery. This was illustrated in particular by the differences in conditions between the mid-level and the community hospital. In the former, caseload midwifery had to be funded by existing resources and was introduced in the aftermath of major organisational restructuring; this made negotiations considerably more difficult. Separate funds for financing caseload midwifery were available at the community hospital, and its introduction came to be seen as measure that strengthened the hospital’s strategic position. These findings corroborate results from recent health service studies stressing that organisational change is highly contingent on local conditions [40–42].

The interviews with key players from both managerial and staff levels provided a rich diversity of perspectives. In addition, management’s assistance with recruiting participants boosted the legitimacy of the study and may have contributed to the high priority given to it by the participants. This enabled us to achieve a high level of variance in caseload models and professional experience of participants. On the other hand, it may also have biased the recruitment of participants and thus the study data. To counter this, we interviewed managers and staff separately and took great care with informing the participants of their rights, including the opportunity to correct in or delete from the transcript. The lively dialogue in the group interviews with frank critique of management and problems related to caseload midwifery lead us to believe that, on the whole, our sampling strategy was advantageous. It was, however, a drawback that we had to limit information about the individual respondents to safeguard their anonymity. This makes it difficult to fully assess bias and puts potential limits to the study’s generalisability.

### Implications for practice and research

Previous studies of organisational change in connection with introducing caseload midwifery [10, 11, 17] have tended to focus on the impact of specific organisational factors. In contrast, we adopt a broader perspective to highlight the complexity of these processes and the potential of the independence of professional and organisational projects. The practical implications of our findings are firstly, that management should take account of the complexity of organisational change processes. Midwives and management engage in formal and informal negotiations, and they also have a wide range of different and sometimes conflicting interests. Secondly, management has an important role in activating staff and management resources in resolving the naturally occurring conflicts in these processes.

Organisational studies of midwifery or other female-dominated professions provide an opportunity for analysing the deep-seated interdependence of professional and organisational projects and for identifying the key dimensions of this interdependence. The literature argues that the close interplay between professional and organisational projects primarily reflects the effects of recent reforms in public health services, which have drawn on a variety of control mechanisms, primarily at the organisational level [30, 31]. However, as argued above, this has been a salient feature of female-dominated professions such as midwifery. Its implications are poorly understood; existing studies mainly discuss the importance of gender in (formerly) male-dominated professions [43–45]. Our analysis has suggested that the deep-rooted interdependence centres on midwives and managers pursuing/drawing on both professional and organisational interests/resources. As a consequence, not only managers are in the mind of professionals [26, 31], but also professionals are in the minds of managers.

## Conclusions

While there is a growing interest in midwife-led continuity models of care, little is known about the processes of organisational change that are involved. Our study of caseload midwifery has focused on the hospital as a shared organisational platform for midwives’ and management’s negotiations on the introduction and development of caseload midwifery in three Danish hospitals.

Our analysis has shown that organisational change in caseload midwifery emerged as a highly interdependent interplay between complex organisational and professional projects: negotiations dominated, midwives and management engaged in both types of projects, and the resolution of tensions was supported by all parties. The interplay was further complicated by ward midwives’ role as a third party. Two major implications for practice are that the complexity of organisational change must be taken into account and that midwives’ and managers’ resources for conflict resolution are vital.

## References

[1] Baker SR, Choi PYL, Henshaw CA, Tree J (2005). “I felt as though I’d been in jail”: Women’s experiences of maternity care during labour, delivery and the immediate postpartum. Fem Psychol..

[2] Kirkham M (2004). Choice and Bureaucracy. Informed Choice in Maternity Care.

[3] Walsh D (2006). Subverting the assembly-line: Childbirth in a free-standing birth centre. Soc Sci Med..

[4] Bai J, Gyaneshwar J, Bauman A (2008). Models of antenatal care and obstetric outcomes in Sydney South West. Aust N Z J..

[5] McLachlan H, Forster D, Davey M, Farrel T, Gold L, Biró M (2012). Effects of continuity of care by a primary midwife (caseload midwifery) on caesarean section rates in women of low obstetric risk: the COSMOS randomised controlled trial. BJOG..

[6] Rowley M, Hensley M, Brinsmead M, Wlodarczyk J (1995). Continuity of care by a midwife team versus routine care during pregnancy and birth: a randomised trial. Med J Aust..

[7] Sandall J, Soltani H, Gates S, Shennan A, Devane D. Midwife-led continuity models versus other models of care for childbearing women. The Cochrane Library. 2013;8. 10.1002/14651858.CD004667.pub3 23963739

[8] Waldenström U, Brown S, McLachlan H, Forster D, Brennecke S (2000). Does team midwife care increase satisfaction with antenatal, intrapartum, and postpartum care? A randomized controlled trial. Birth..

[9] Forster DA, Newton M, McLachlan H, Willis K (2011). Exploring implementation and sustainability of models of care: can theory help?. BMC Public Health.

[10] Walker S, Moore H, Eaton A (2004). North Queensland midwives’ experience with a team model of midwifery care. Aus J Midwifery..

[11] Hartz D, White J, Lainchbury K, Gunn H, Jarman H, Welsh A (2012). Australian maternity reform through clinical redesign. Aust Health Rev..

[12] Bourgeault I, Fynes M (1997). Integrating lay and nurse-midwifery into the US and Canadian health care systems. Soc Sci Med..

[13] Sandall J (1996). Continuity of midwifery care in England: a professional project?. Gend Work Organ..

[14] Wrede S. Decentering care for mothers. The politics of midwifery and the design of Finnish maternity services. Åbo: Åbo Akademi University Press; 2001.

[15] Benoit C, Shroff FM (1997). Professionalizing Canadian midwifery: sociological perspectives. The new midwifery. Reflections on renaissance and regulation.

[16] Homer C, Brodie P, Leap N, Homer C, Brodie P, Leap N (2005). Getting started: what is midwifery continuity of care. Midwifery continuity of care: a practical guide.

[17] Andrews S, Brown L, Bowman L, Price L, Taylor R (2006). Caseload midwifery: a review. Midwifery Matters..

[18] Tracy SK, Hartz D, Nicholl M, McCann Y, Latta D (2005). An integrated service network in maternity – the implementation of a midwifery-led unit. Aust Health Rev..

[19] McCourt C (2006). Supporting choice and control? Communication and interaction between midwives and women at the antenatal booking visit. Soc Sci Med..

[20] Henriksson L, Wrede S, Burau V (2006). Understanding professional projects in welfare service work: revival of old professionalism?. Gend Work Organ..

[21] Benoit C, Wrede S, Bourgeault I, Sandall J, DeVries R, Teijlingen ER (2005). Understanding the social organisation of maternity care systems: midwifery as a touchstone. Sociol Health Illn..

[22] Declercq E, DeVries R, Viisainen K, Salvesen HB, Wrede S, De Vries R, Benoit C, Van Teijlingen ER, Wrede S (2001). Where to give birth? Politics and the place of birth. Birth by design. Pregnancy, maternity care and midwifery in North America and Europe.

[23] Committee on Quality of Health Care in America, Institute of Medicine (2001). A New Health System for the 21st Century.

[24] World Health Organisation (2000). The World Health report 2000 – Health systems: Improving performance.

[25] French M, Miller F (2012). Leveraging the “living laboratory”: on the emergence of the entrepreneurial hospital. Soc Sci Med..

[26] Kuhlmann E, Burau V, Correia T, Lewandowski R, Lionis C, Noordegraad M (2013). “A manager in the minds of doctors:” a comparison of new modes of control in European hospitals. BMC Health Serv Res..

[27] Reat T, Hinings C (2009). Managing the rivalry of competing institutional logics. Organ Stu..

[28] Waring J, Currie M (2009). Managing expert knowledge: organizational challenges and managerial futures of the UK medical profession. Organ Stu..

[29] Muzio D, Brock D, Suddaby R (2013). Professions and institutional change: towards an institutionalist sociology of the professions. J Manag Stud.

[30] Muzio D, Kirkpatrick I (2011). Professions and organizations – a conceptual framework. Curr Sociol..

[31] Noordegraaf M (2011). Risky business: how professionals and professional fields (must) deal with organizational issues. Organ Stu..

[32] Cliff H (1993). Jordemoderliv.

[33] Jessen MH (2008). Naturfleksibilitetens vilkår i jordemoderkonsultationen. En autoetnografisk tilgang til mødet mellem jordemoderen og den gravide kvinde.

[34] Kjeldset A-M (2008). Hvorfor gør vi, som vi gør?. Tidsskrift for Jordemødre..

[35] May C, Finch T (2009). Implementation, embedding, and integration: an outline of normalization process theory. Sociology.

[36] Sandall J, Bourgeault I, Meijer W, Schüecking B, De Vries R, Benoit C, Van Teijlingen ER, Wrede S (2001). Deciding who cares: winners and loosers in the late twentieth century. Birth by design. Pregnancy, maternity care and midwifery in North America and Europe.

[37] Sandall J, Benoit C, Wrede S, Murray S, Van Teijlingen E, Westafall R (2009). Social service professional or market expert?: Maternity care relations under neoliberal healthcare reform. Curr Sociol..

[38] Thomas G (2011). A typology for the case study in social science following a review of definition, discourse, and structure. Qual Inq..

[39] Braun V, Clarke V (2006). Using thematic analysis in psychology. Qual Res Psychol..

[40] Craig P, Dieppe P, Macintyre S, Michie S, Nazareth I, Pettigrew M (2008). Developing and evaluating complex interventions: new guidance.

[41] Damschroder L, Aron D, Keith R, Kirsh S, Alexander J, Lowery J (2009). Fostering implementation of health services research findings into practice: a consolidated framework for advancing implementation science. Implement Sci..

[42] Øvretveit J (2010). Understanding the conditions for improvement: research to discover which context influences affect improvement success. BMJ Qual Saf..

[43] Bolton SC, Muzio D (2007). Can’t live with “em; can”t live without ‘em. Gendered segmentation in the legal profession. Sociology..

[44] Bolton S, Muzio D (2008). The paradoxical processes of feminization in the professions: the case of established, aspiring and semi-rofessions. Work Employ Soc..

[45] Muzio D, Tomlinson J (2012). Editorial: researching gender, inclusion and diversity in contemporary professions and professional service organizations. Gend Work Organ..

